# Disparity in Access to Oncology Precision Care: A Geospatial Analysis of Driving Distances to Genetic Counselors in the U.S.

**DOI:** 10.3389/fonc.2021.689927

**Published:** 2021-06-16

**Authors:** Mathias M. J. Bellaiche, Winnie Fan, Harold John Walbert, Egan H. McClave, Bradley L. Goodnight, Fred H. Sieling, Rebekah A. Moore, Weilin Meng, Christopher M. Black

**Affiliations:** ^1^ Guidehouse Inc., McLean, VA, United States; ^2^ Precision Medicine & Nurse Education, AstraZeneca Pharmaceuticals, Gaithersburg, MD, United States; ^3^ Center for Observational and Real-World Evidence (CORE), Merck & Co., Inc., Kenilworth, NJ, United States

**Keywords:** Genetic counseling, health access, social determinants of health, geographic information system, cancer care, precision medicine, *BRCA*, healthcare equity

## Abstract

In the US, the growing demand for precision medicine, particularly in oncology, continues to put pressure on the availability of genetic counselors to meet that demand. This is especially true in certain geographic locations due to the uneven distribution of genetic counselors throughout the US. To assess these disparities, access to genetic counselors of all specialties is explored by geography, cancer type, and social determinants of health. Geospatial technology was used to combine and analyze genetic counselor locations and cancer incidence at the county level across the US, with a particular focus on tumors associated with *BRCA* mutations including ovarian, pancreatic, prostate and breast. Access distributions were quantified, and associations with region, cancer type, and socioeconomic variables were investigated using correlational tests. Nationally, in 2020, there were 4,813 genetic counselors, or 1.49 genetic counselors per 100,000 people, varying between 0.17 to 5.7 per 100,000 at the state level. Seventy-one percent of U.S. residents live within a 30-minute drive-time to a genetic counselor. Drive-times, however, are not equally distributed across the country – while 82% of people in metropolitan areas are 30 minutes from a genetic counselor, only 6% of people in nonmetro areas live within 30 minutes’ drive time. There are statistically significant differences in access across geographical regions, socioeconomics and cancer types. Access to genetic counselors for cancer patients differs across groups, including regional, socioeconomic, and cancer type. These findings highlight areas of the country that may benefit from increased genetic counseling provider supply, by increasing the number of genetic counselors in a region or by expanding the use of telegenetics a term used to describe virtual genetic counseling consults that occur *via* videoconference. Policy intervention to allow genetic counselors to bill for their services may be an effective route for increasing availability of genetic counselors’ services However, genetic counselors in direct patient care settings also face other challenges such as salary, job satisfaction, job recognition, overwork/burnout, and appropriate administrative/clinical support, and addressing these issues should also be considered along with policy support. These results could support targeted policy reform and alternative service models to increase access to identified pockets of unmet need, such as telemedicine. Data and analysis are available to the public through an interactive dashboard^1^.

## Introduction

Advances in genomic research, new testing technologies, increased use of electronic medical records, and general public interest have led to the expansion of precision medicine, which is an approach to patient care that allows doctors to select treatments that are most likely to help patients based on a genetic understanding of their disease (1). Precision medicine offers the potential to improve health outcomes by allowing providers and patients to select treatments most likely to be effective considering an individual’s genetic, environmental, and lifestyle traits (1). The expansion of genetic and genomic testing has increased demand for providers along the oncology patient journey, particularly on genetic counselors, to educate patients and promote informed decision-making (2). Previous research has shown that current and forecast demand for genetic counselors exceeds supply, that the spatial distribution of genetic counselors is variable across the southern United States, and that local access to a genetic counselor is related to social determinants of health (SDoH) such as race or household income (2–4). To measure differences in access and health system equity, it is imperative to describe the spatial patterns of provider access for cancer patients, as well as how access is affected by factors such as socioeconomics, geographical region and cancer type (2, 5).

Genetic counseling is particularly relevant for cancer care, where genetic counselors meet with patients to advise on risk of hereditary cancer syndromes and discuss cancer screening, risk-reduction, and treatment options. National consensus guidelines recommend genetic counseling for patients with a personal and/or family history of cancer that is suggestive of a hereditary cancer syndrome (6), highlighting the pivotal role played by these providers for precision oncological care. The National Comprehensive Cancer Network recommends germline *BRCA* testing for individuals with a personal and/or family history of ovarian or pancreatic cancer, and a personal and/or family history of breast or prostate cancer as long as other criteria are met (age of diagnosis, number of family members with associated cancers, ethnicity, etc.) (6). These recommendations represent numerous patients and a source of stress on the medical system. Although the COVID-19 pandemic has caused a dramatic transition to telework and telehealth, significant barriers exist to the expansion of such alternative service delivery models for virtual genetic counseling, such as Medicare policy that does not recognize genetic counselors as providers eligible for reimbursement of any services, virtual or in-person (7). Furthermore, 26 states require licensure for genetic counselors, but the lack of license reciprocity options can limit the ability of genetic counselors to practice across state lines when offering telegenetics or virtual genetic counseling appointments (8). As such, access to in-person genetic counselors remains critical to understand.

Geographical information systems (GIS) offer a method to understand spatial distributions of cancer patients, including subsets of those with cancers associated with *BRCA* mutational status such as breast, ovarian, prostate and pancreatic (9), and providers in the context of local factors that may affect health outcomes. This technology has been applied to medical contexts, including linking hotspots of kidney disease with water contamination, partitioning the United States into regions based on SDoH and describing distributions of vaccine providers relative to patients (10–12). The objective of the current analysis was to leverage GIS methods to map and quantify the spatial distributions of genetic counselors at the national level, incorporate data on disease burden and population characteristics to measure real demand for genetic counseling, quantify the degree of supply-demand match *via* per-capita access metrics, derive time to travel to genetic counselors across the country, and investigate differences in these distributions according to SDoH.

## Materials and Methods

Geospatial methods were used to understand the relationship between genetic counselor locations, patient population locations, and SDoH in the US. Data were collected along three categories: location of genetic counselors working in direct patient care, incidences of cancers (for all cancer subtypes and those specifically associated with *BRCA* mutations: breast, ovarian, prostate, and pancreatic cancers) and socioeconomic variables.

Data on genetic counselors in the United States were extracted from three sources: the Centers for Medicare & Medicaid Services (CMS) National Plan and Provider Enumeration System (NPPES) National Provider Identifier (NPI) registry, and the public-facing member directories of both the American Board of Genetic Counseling (ABGC) and the National Society of Genetic Counselors (NSGC) (see **Supplement**). All data were obtained between May and July of 2020. Although NSGC and ABGC datasets included information on provider specialty, the NPPES dataset did not include that information. The present analysis did not stratify by provider specialty, such as cancer genetics.

The office addresses of the genetic counselors (as self-reported in the public-facing membership directories or present in the business practice location address field of NPPES) were converted into latitude and longitude using Google Maps Geocoding. Individual datasets were cleaned and reconciled into a registry of 4,813 unique genetic counselors across the 50 states and the District of Columbia (See **Supplemental Figure 3** for count of genetic counselors by data source).

Cancer incidence rates for the years 2013-2017, the latest years for which incidence data are available, were downloaded from the Centers for Disease Control and Prevention (CDC) United States Cancer Statistics (USCS) program and filtered to county-level statistics on total (sum of all cancer types), breast, ovarian, pancreatic and prostate cancer. Note that USCS does not contain data on Kansas or Minnesota incidence rates, as those states prohibit release of county-level data (state-level data were available and incorporated into relevant analyses).

County-level demographic data were downloaded from the American Community Survey (ACS) 2018 5-year estimates, including total population, median household income, median age (for all residents and separately for men and women), and estimates of population stratified by sex, race, Hispanic origin, highest educational attainment for those aged at least 25 years, employment status for those aged at least 16 years (either civilian or armed forces), and health insurance coverage (public, private, and either). Counties were classified into metropolitan and nonmetropolitan categories using Rural-Urban Continuum Codes downloaded from the Economic Research Service at the United States Department of Agriculture^2^.

Patient access to care, in this study meaning patient’s physical proximity to genetic counselors, was calculated at the state level by taking the weighted median of county-level drive-time to the closest provider, weighted by cancer incidence rate, for all cancer types or by each of four *BRCA*-associated tumor sites. County shapefiles for GIS were downloaded from the US Census Bureau and used to categorize counties into two groups, depending on whether any genetic counselor latitude/longitude point fell within a county polygon. The distance between each county and the closest genetic counselor was determined by calculating the Haversine distance (surface distance between two points on a sphere) between population-weighted county centroids (from the US Census Bureau) and all providers. Population weighted-county centroids reflect population distributions within a county, in contrast to geographic centroids. The Mapbox Matrix API was used to calculate drive-times to the nearest 20 genetic counselors for each county to render the calculation computationally feasible on standard machines.

Hypothesis tests were conducted for differences in SDoH between counties in the two access groups and for differences in access to care between Census regions, cancer types, and combinations of regions and cancer types. SDoH variables considered were age, sex, race, ethnicity, household income, employment, health insurance coverage and education. An analogous analysis of virtual state-level access and correlations with SDoH was also performed (see **Supplemental**). The Wilcoxon rank-sum test was used to investigate access differences, the Kruskal-Wallis test was used to investigate overall differences in access, the pairwise Wilcoxon test was used to investigate pairwise differences in access, and *p*-values were Bonferroni-corrected to account for multiple comparisons. Statistical analyses were performed using the R Statistical Software (13).

## Results

**Figure 1:**
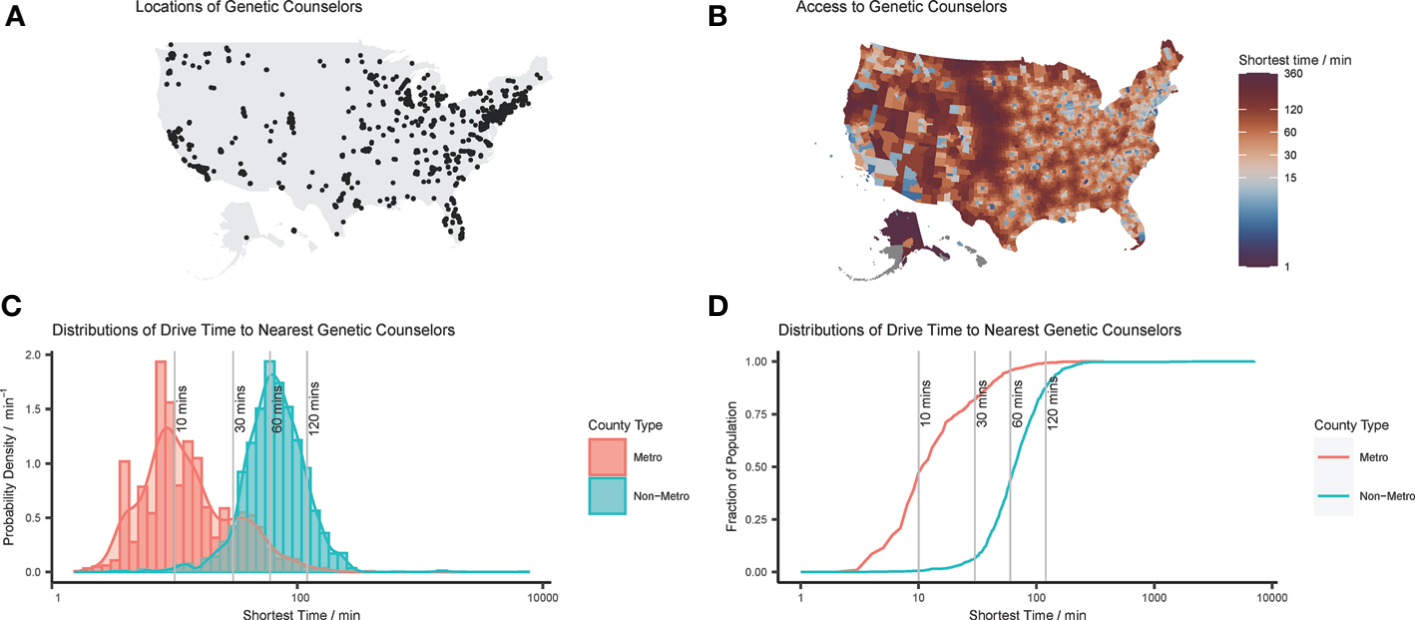
Distributions of genetic counselors in the United States: **(A)** point locations of genetic counselors reconciled from disparate data sources; **(B)** mapped distribution of county-level drive-time to closest genetic counselor; **(C)** histogram and probability density function of county-level drive-time to nearest genetic counselor; and **(D)** cumulative density function of county-level drive-time to nearest genetic counselor. Note that in **(C, D)** the shortest drive-times to a genetic counselor are plotted on log axes, and that these values are population-weighted to take into account relative county populations.

A total of 4,813 genetic counselors were identified, or 1.49 genetic counselors per 100,000 people nationally. Mississippi demonstrated the lowest rate of 0.17 genetic counselors per 100,000 people, and Washington, D.C. had the highest rate of 5.7 per 100,000. **Figures 1A, B** show maps of genetic counselor locations and county-level drive-time to the nearest provider, with a median drive-time of 60.3 minutes (range 1.7 – 7102.4, IQR 57.3). These spatial distributions show that genetic counselors tend to cluster together especially in urban areas, resulting in varied access to care. Distributions in **Figures 1C, D** show that metropolitan counties have systematically shorter drive-times to care: weighting by population shows that while 71% of people in the U.S. are within 30 minutes of their nearest genetic counselor, it is 82% for metro residents (median 33 minutes, range 2 – 374, IQR 34), in contrast to 6% for nonmetro residents (median 79 minutes, range 3 – 7102, IQR 56).

**Figure 2:**
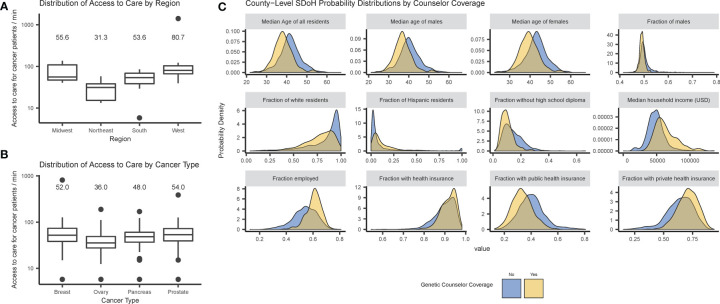
Disparities in access to care by cancer patients and genetic counselor access: **(A)** box plots of access metric by U.S. Census region; **(B)** box plots of access metrics by cancer type; and **(C)** probability densities of county-level SDoH distributions by genetic counselor access. Note that in **(A, B)**, state-level access (defined as the median drive time for a cancer patient to a genetic counselor) is plotted on a log axis, and the medians are provided as labels.

**Figure 2** shows disparities in access to care as correlations between physical proximity to care and geographical region (A) and cancer type (B), or between genetic counselor access and SDoH (C). Distributions of the outcome variables (SDoH or access) show differences by genetic counselor access consistent with differences between metropolitan and nonmetropolitan counties, and similar differences exist when explicitly testing for differences due to metropolitan county status.

Physical access to care significantly differs between regions (*p* = 0.001), with the largest difference between the West and Northeast regions (*p* = 0.006). There are also significant differences in access between different types of incident cancer patients (*p* < 0.001), with the largest between prostate and ovarian cancer (*p* < 0.001; for complete results see **Supplemental Tables 1**, **2**). These tests were repeated using physical distance instead of drive-time with similar results (see **Supplemental**).

Further analysis investigated correlations between SDoH and genetic counselor access, revealing significant differences between counties with and without genetic counselor access in all demographic variables – age, sex, race, Hispanic origin, employment status, health insurance coverage and educational attainment (see **Supplemental Table 3**). The largest effect was for median household income (*p* < 0.001) — counties with a genetic counselor had a median income of $60,000/year while counties without a genetic counselor had median income under $50,000/year.

Virtual access was also investigated, though these analyses were limited by the fact that only NSGC reported data on which members provide telegenetic services. The average state-level virtual access was 2 genetic counselors per 100,000 people (range 0.3 – 10 per 100,000), with genetic counselors assumed to provide services only to residents of their states as reported in the NSGC registry. Note that this calculation does not consider the effects of multi-state licensure, i.e., cases in which a genetic counselor could technically provide services in additional states beyond those self-reported due to lack of licensure. Genetic counselors may provide both in-person and telegenetics from the same location.

## Discussion

The purpose of this study was to evaluate equity of access to genetic counselors at a national level across the United States. The number of genetic counselors per 100,000 people was 1.49, on average, but this belies considerable state-level variability, ranging from 0.17 to 5.7. These findings show systematic differences in access to care between regions, and provide insight for potential policy action, e.g., in the southern United States where overall cancer incidence is relatively high and genetic counselors are relatively few. Furthermore, there are associations between socioeconomic characteristics and access, with counties with genetic counselors being younger, more diverse, with a higher level of education, and with higher incomes, consistent with previous studies and typical for urban locales (4).

The novelty of this study is its national scale and use of GIS to connect disease burden to provider supply, allowing for direct measures of access equity. The method allows for granular identifications of areas with differential access: for example, Williamson County, Tennessee, and Runnels County, Texas, have similar cancer burdens (approximately 435 incident patients per 100,000 annually), but their per-capita access is 0.5 and 38.8 respectively. Underserved patient populations are revealed for further research, e.g., for policy reform to improve access to either in-person or virtual genetic counseling (also known as telegenetics). For example, Gloucester County, New Jersey had among the poorest mismatches between genetic counselor supply and demand, with one genetic counselor for a population of nearly 300,000 people despite having an annual cancer incidence rate of 542.2 per 100,000, the fifth highest rate across all U.S. counties. To communicate these results more directly to the public an interactive dashboard is available that includes maps of genetic counselor locations and visuals of accessibility distributions^3^.

Measurements of driving time required to see a genetic counselor are particularly telling, showing that most metropolitan residents live within one hour of a genetic counselor, but most nonmetropolitan residents do not. Previous research has shown that patients that are more than an hour from care are less likely to access the health system and are associated with poorer health outcomes (14, 15). Long drive times to a provider have negative implications for patient adherence to standard-of-care recommendations and are therefore important for continuity of care. Telegenetics has the potential to increase access to genetic counselors for these patients who do not live within a reasonable driving distance to a genetic counselor, or even for individuals who are on a long waitlist to see an in-person genetic counselor.

The Supplemental Information includes analogous analysis of available telegenetic access data, but these are complicated by nuances such as differences in state licensure requirements and provider eligibility in payer reimbursement rules, which are beyond the scope of this study as no robust single data source exists to capture these complications. Virtual genetic counseling care has potential to increase patient access to genetic counselors, both in-state with a far driving distance as outlined above or supporting access across state lines as drive time and distance is no longer a barrier. Currently, only 26 states require licensure (8) to practice and other states only require board certification (so they could be accessible to all 3077 genetic counselors identified in the ABGC data). Furthermore, some states demonstrated a high degree of disparity between the number of in-state genetic counselors versus virtual genetic counselors. For example, Wyoming had only one in-state genetic counselor but up to 58 virtual genetic counselors practicing in the state. Expanding such alternative service delivery models would, however, require policy shifts such as legislative changes to allow genetic counselors to be reimbursed for virtual care through Medicare for their services of both in-person and virtual appointments (7).

Limitations of this study include reconciling non-standardized genetic counselor data from NSGC, ABGC, and NPPES which included manual review and may have introduced error. A sensitivity analysis, however, showed no changes in statistical results when the genetic counselor data were subset by source (see **Supplemental**). As any genetic counselor is able to provide services within any medical specialty without the need for formal accreditation and specialty was only available in one of the data sources (NSGC), data were not stratified by self-reported specialty. As a result, access to genetic counselors who exclusively specialize in cancer care may be lower than what is presented in these results. The numbers of genetic counselors are overestimated as registries include those in private third-party labs, academia or industry that do not see patients. Furthermore, the data represents the genetic counselor population at a single point in time (May 2020 - July 2020) and may include individual genetic counselors that have stopped seeing patients since that time, but does not include genetic counselors who have graduated from genetic counseling training programs. While the data were collected during the beginning of the COVID-19 pandemic, the dataset only included genetic counselors who noted they provided telegenetics in the NSGC directory and did not include any genetic counselors who began utilizing telegenetics during the COVID-19 pandemic, but did not update their NSGC public-facing profile to reflect this change in their practice. In addition, the dataset likely does not capture the full volume of genetic counselors who currently provide telegenetic counseling today as it has become more common as the COVID-19 pandemic continued.

Distance calculations used population-weighted county centroids and these locations could be misleading in edge cases such as multiple islands making up one county. Furthermore, distance is only one aspect of access to care, and other aspects, such as reimbursement policy or licensure requirements, should be considered in future research. In the instances where these points are not accessible *via* driving, the nearest accessible location was used. The drive-time analysis did not consider traffic patterns or time of day.

Lastly, this approach is limited by the lack of causal analysis. Socioeconomic variables, such as household income, may be associated with region and metropolitan/rural status, but these effects have not been controlled for here because reliable data at the county-level were not available.

This study represents a first step in understanding patients’ ability to receive genetic counseling at the country-level, by comprehensively mapping access to in-person care using three major national data sources. Future work should build on this by analyzing access to virtual care more closely, especially considering the rising importance of this delivery channel due to the COVID-19 pandemic. Such an analysis would need to address complexities with differences in state-level licensure requirements and incorporate data on clinician time and capacity.

Future work could also refine measures of access to include public transport and could incorporate health outcomes and measures of provider utilization to investigate the effects of access to care. Additional analyses could establish how these determinants combine to enable or block access and utilization. Finally, forming partnerships between health researchers and professional societies such as NSGC or ABGC may improve data sharing, reduce data integration issues, and allow for deeper analysis that includes patient load, appointment wait times, and workforce trends.

Ultimately these results demonstrate systematic differences in proximity to genetic counseling, illustrating disparity in access to genetic counselors throughout the US. Such findings establish GIS as a powerful tool for investigating the ability of patients to physically interact with the healthcare system and provide implications for policy interventions to expand access, especially in regions with a high unmet need and few genetic counselors.

## Data Availability Statement

Publicly available datasets were analyzed in this study. This data can be found here: NSGC https://www.nsgc.org/page/find-a-gc-search, USCS https://www.cdc.gov/cancer/uscs/dataviz/download_data.htm, ABGC https://www.abgc.net/about-genetic-counseling/find-a-certified-counselor/, and NCI SEER https://seer.cancer.gov/data/access.html.

## Author Contributions

MB, BG, FS, and CB: conceptualization, methodology, writing—original draft, writing—review and editing, supervision, and project administration. WF, HW, and EM: data curation, formal analysis, investigation, visualization, writing—original draft, and writing—review and editing. RM and WM: conceptualization, methodology, writing—original draft, and writing—review and edition. All authors contributed to the article and approved the submitted version.

## Funding

This study was funded by Merck & Co., Inc., Kenilworth, N.J., U.S.A and AstraZeneca Pharmaceuticals.

## Conflict of Interest

Authors MB, WF, HW, EM, BG, and FS were employed by the company Guidehouse, Inc. Author RM was employed by AstraZeneca Pharmaceuticals. Authors CB and WM were employed by Merck & Co., Inc., Kenilworth, N.J., U.S.A. MB and RM are stockholders of AstraZeneca Pharmaceuticals. CB and WM are stockholders of Merck & Co., Inc. Merck & Co., Inc and AstraZeneca Pharmaceuticals co-develop Lynparza (olaparib), a drug used to treat certain cancers of interest to this study.

The authors declare that this study received funding from Merck & Co., Inc., Kenilworth, N.J., U.S.A and AstraZeneca Pharmaceuticals. The funder had the following involvement with the study: conceptualization, methodology, writing—original draft, writing—review and editing, supervision and project administration.

## Footnotes

2 Downloaded from https://www.ers.usda.gov/data-products/rural-urban-continuum-codes.aspx.

3 Interactive dashboard available at https://gisgeneticcounselor.shinyapps.io/CounselorExplorer/.

## Supplementary Material

Supplementary Figure 1 Provenance of genetic counselor data: Venn diagram of numbers of unique genetic counselors extracted from each source of provider information.

Supplementary Figure 2 Access to care by patients with *BRCA*-associated cancers by cancer type and U.S. region: box plots of access metric by combination of U.S. Census region and cancer type. Note that the state-level access to care (defined as the median drive time for a cancer patient to a genetic counselor) is plotted on a log axis.

Supplementary Figure 3 Virtual genetic counselor access: histogram of state-level per-capita numbers of virtual counselors and measures of central tendency.

Supplementary Figure 4 State-level SDoH: probability densities of state-level SDoH distributions by mean-split per-capita virtual genetic counselor density.
