# Supplementary figures and images for: Disparity in Access to Oncology Precision Care: A Geospatial Analysis of Driving Distances to Genetic Counselors in the U.S
Source: Front Oncol. 2021 Jun 16;11:689927. doi: 10.3389/fonc.2021.689927 (PMC8242948; doi:10.3389/fonc.2021.689927)

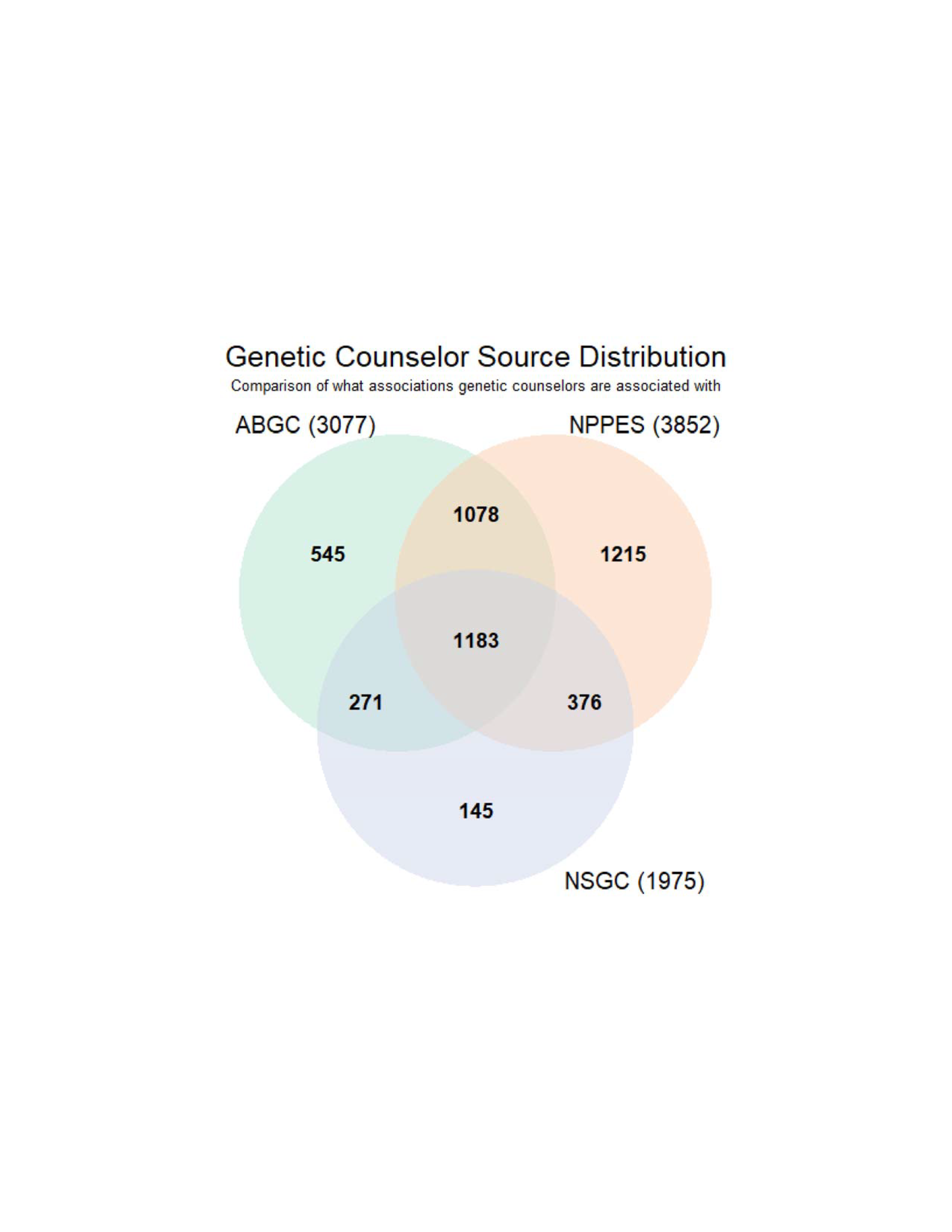

Supplement: Supplementary Figure 1 — Provenance of genetic counselor data: Venn diagram of numbers of unique genetic counselors extracted from each source of provider information. [file Image_1.tif]

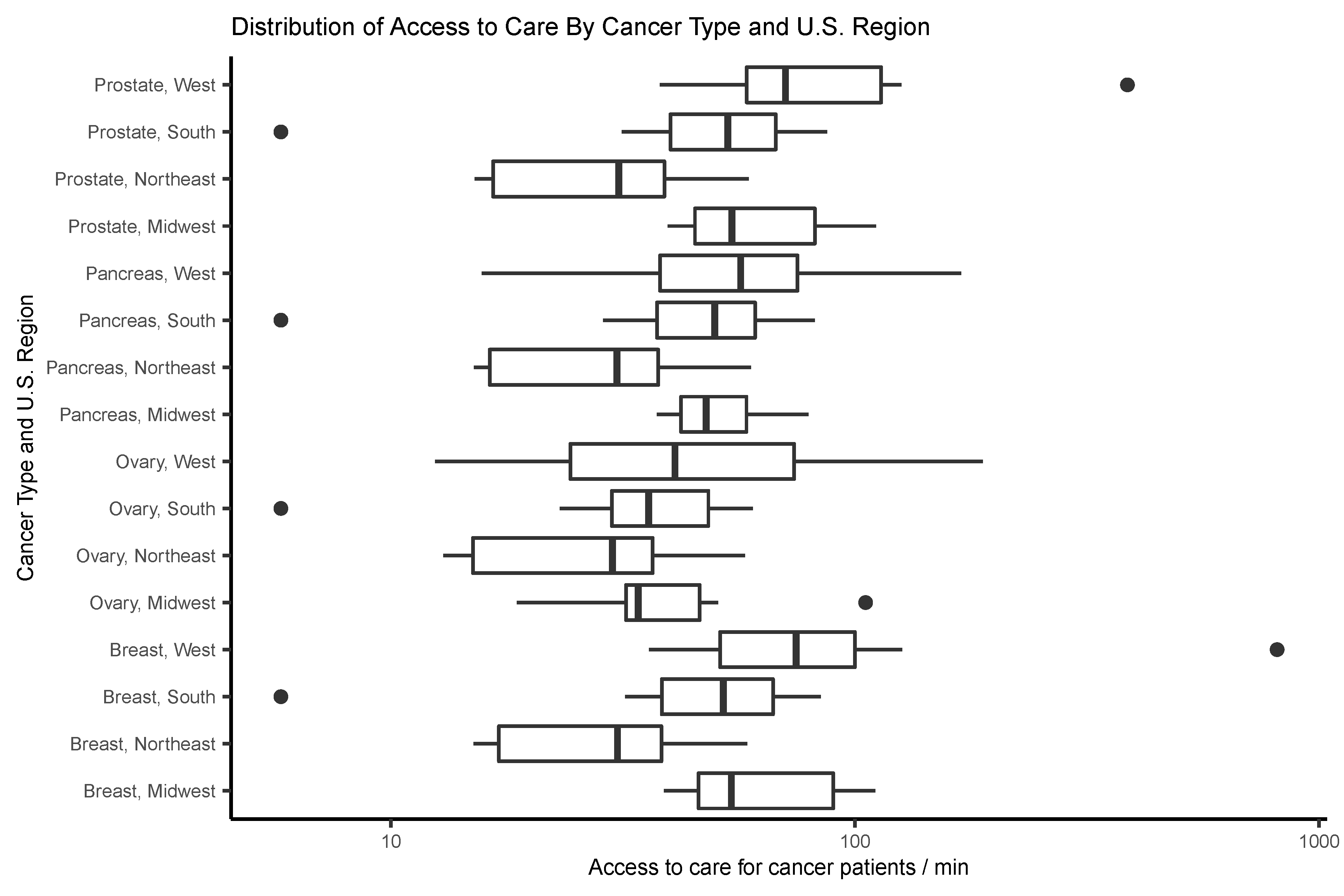

Supplement: Supplementary Figure 2 — Access to care by patients with BRCA-associated cancers by cancer type and U.S. region: box plots of access metric by combination of U.S. Census region and cancer type. Note that the state-level access to care (defined as the median drive time for a cancer patient to a genetic counselor) is plotted on a log axis. [file Image_2.tif]

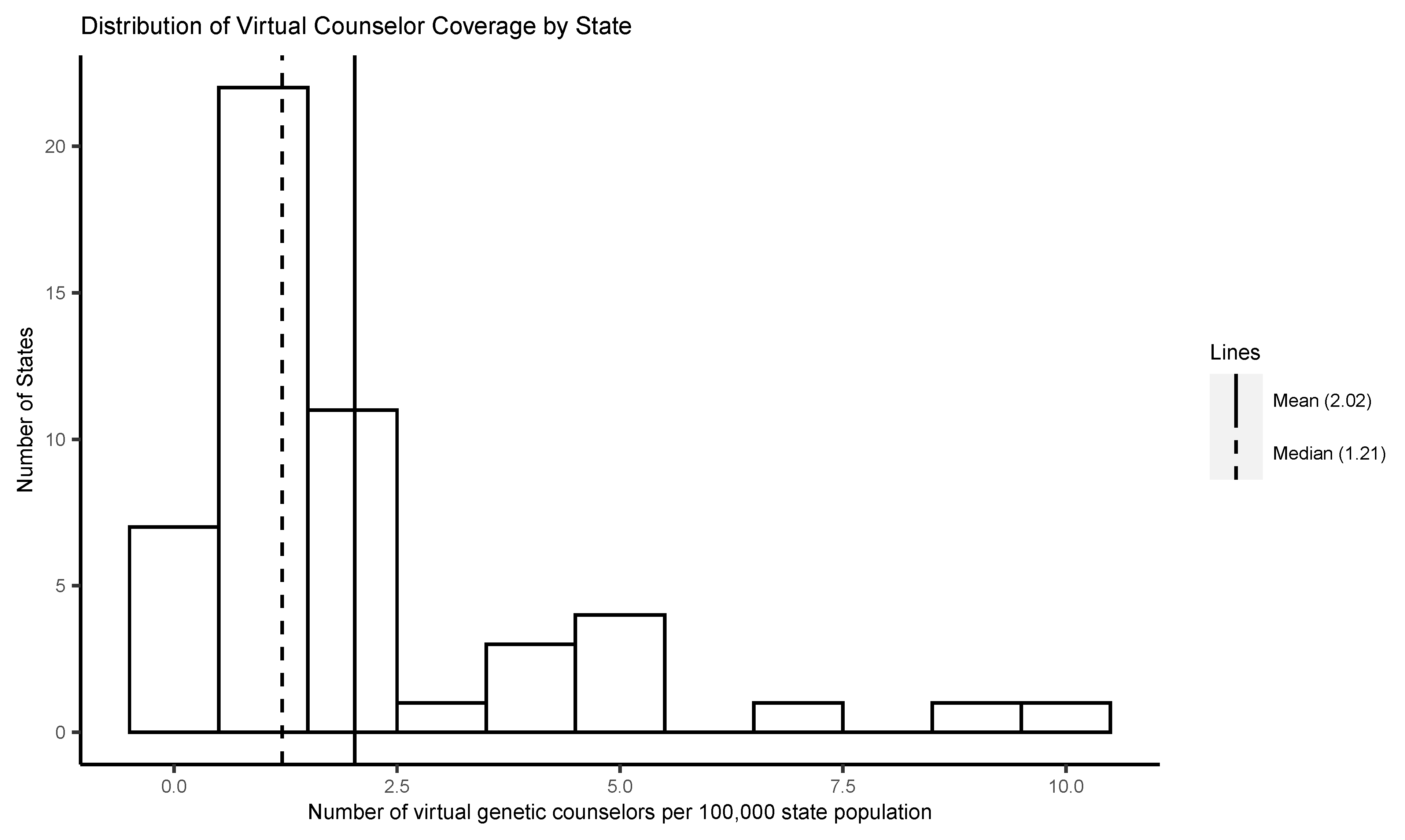

Supplement: Supplementary Figure 3 — Virtual genetic counselor access: histogram of state-level per-capita numbers of virtual counselors and measures of central tendency. [file Image_3.tif]

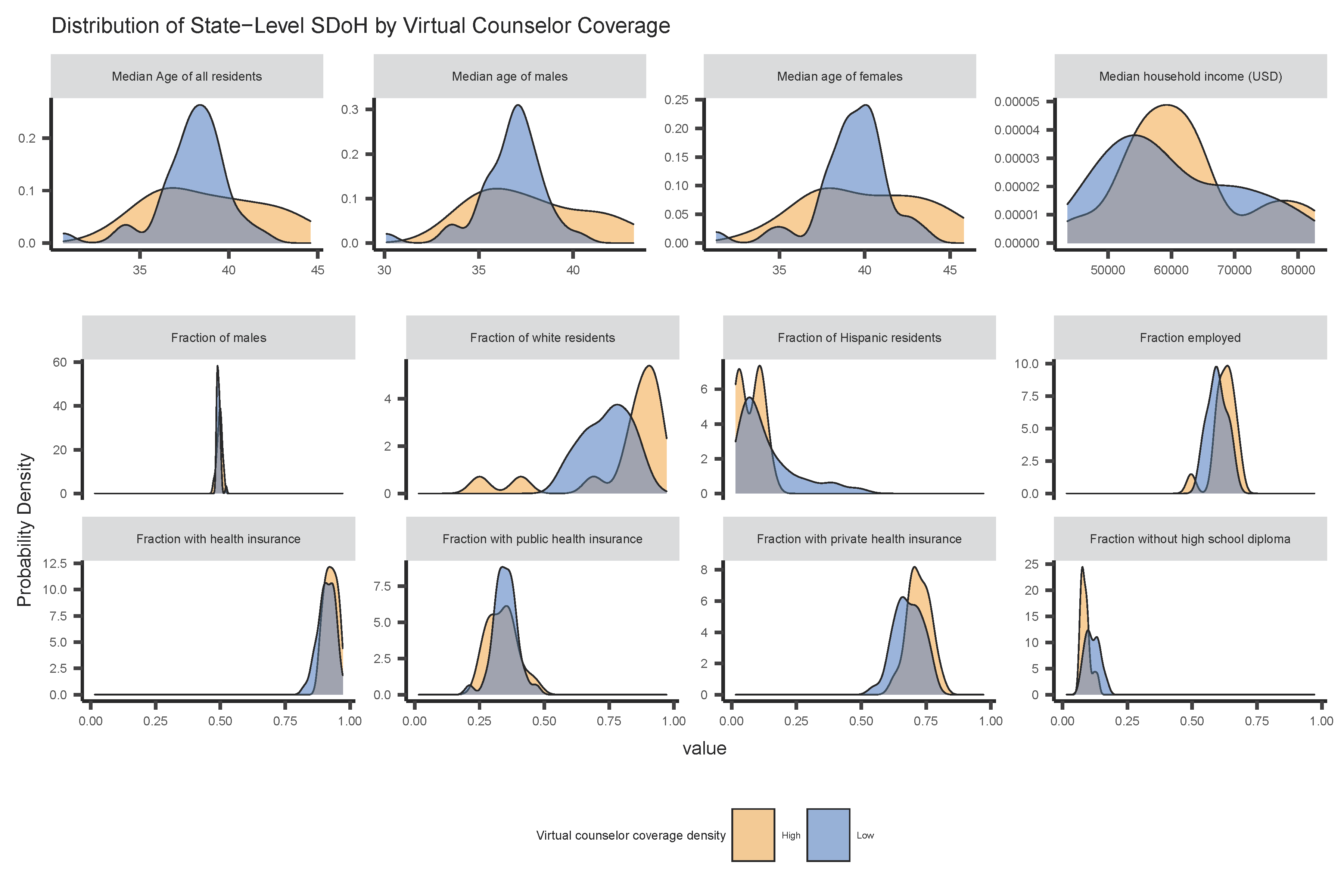

Supplement: Supplementary Figure 4 — State-level SDoH: probability densities of state-level SDoH distributions by mean-split per-capita virtual genetic counselor density. [file Image_4.tif]
