# Supplementary material for: Disparity in Access to Oncology Precision Care: A Geospatial Analysis of Driving Distances to Genetic Counselors in the U.S
Source: Front Oncol. 2021 Jun 16;11:689927. doi: 10.3389/fonc.2021.689927 (PMC8242948; doi:10.3389/fonc.2021.689927)
Supplement: Supplementary file 5 [file Table_1.pdf]

Table 1. Median and IQR of genetic counselor access to care (state-level median of drive-times to the nearest genetic counselor, weighted by cancer incidence rates) for cancer patients by U.S. region

Region	Median of access / mins	IQR of access / mins
**Midwest**	55.6	61.7
**Northeast**	31.3	22.8
**South**	53.6	30.0
**West**	80.7	37.2
