# Supplementary material for: Disparity in Access to Oncology Precision Care: A Geospatial Analysis of Driving Distances to Genetic Counselors in the U.S
Source: Front Oncol. 2021 Jun 16;11:689927. doi: 10.3389/fonc.2021.689927 (PMC8242948; doi:10.3389/fonc.2021.689927)
Supplement: Supplementary file 6 [file Table_2.pdf]

Table 2. Median and IQR of access to care (state-level median of drive-times to the nearest genetic counselor, weighted by cancer incidence rates) for patients with *BRCA*-associated cancers by cancer type

Cancer type	Median of access / mins	IQR of access / mins
Breast	52.0	35.3
Ovary	35.5	21.2
Pancreas	48.5	23.4
Prostate	53.2	33.3
