# Supplementary material for: Disparity in Access to Oncology Precision Care: A Geospatial Analysis of Driving Distances to Genetic Counselors in the U.S
Source: Front Oncol. 2021 Jun 16;11:689927. doi: 10.3389/fonc.2021.689927 (PMC8242948; doi:10.3389/fonc.2021.689927)
Supplement: Supplementary file 7 [file Table_3.pdf]

Table 3. County-level SDoH by genetic counselor access groups (in-person vs. without in-person). Note that dispersion is represented by the inter-quartile range, which is not sensitive to sample size and hence not a reflection of population variance. (*: >95% confidence; **: >99% confidence; *:>99.9% confidence, N.S.: < 90% confidence)**

Characteristic (median by county, with IQR in parentheses)		Counties with in-person genetic counselor access [1] *N = 435 counties*	Counties without in-person genetic counselor access *N = 2,785 counties*	Wilcoxon Rank Sum test (Bonferroni corrected *p*-value) *N = 12 tests*
**Median age of county residents / yr**	*All*	38.1 (5.6)	41.6 (5.9)	*** (<0.001)
	Male	36.8 (5.1)	40.1 (6.1)	*** (<0.001)
	Female	39.4 (6.0)	43.2 (6.0)	*** (<0.001)
**Fraction of county population that is male**		0.49 (0.01)	0.50 (0.02)	*** (<0.001)
**Fraction of county population that is white**		0.8 (0.2)	0.9 (0.2)	*** (<0.001)
**Fraction of county that is of Hispanic origin**		0.08 (0.13)	0.04 (0.07)	*** (<0.001)
**Median household income in county / $**		59,943 (20,899)	48,235 (14,557)	*** (<0.001)
**Fraction of people in county that are ages 16+ and employed**		0.61 (0.07)	0.54 (0.12)	*** (<0.001)
**Fraction of people in county with health insurance coverage**	*Any*	0.92 (0.05)	0.91 (0.07)	*** (<0.001)
	Public	0.33 (0.10)	0.40 (0.12)	*** (<0.001)
	Private	0.71 (0.12)	0.65 (0.15)	*** (<0.001)
**Fraction of people in county that are ages 25+ and without a high school degree**		0.10 (0.05)	0.13 (0.09)	*** (<0.001)

[1] *N*: Number of samples, *IQR*: Interquartile range
