# Supplementary material for: Disparity in Access to Oncology Precision Care: A Geospatial Analysis of Driving Distances to Genetic Counselors in the U.S
Source: Front Oncol. 2021 Jun 16;11:689927. doi: 10.3389/fonc.2021.689927 (PMC8242948; doi:10.3389/fonc.2021.689927)
Supplement: Supplementary file 8 [file Table_4.pdf]

Table 4. Median and IQR of access to care for *BRCA*-associated cancer types in different U.S. regions

Region	Cancer type	Median of access/ mins	IQR of access /mins
Midwest	Breast	54.1	44.9
	Ovary	34.1	14.7
	Pancreas	47.8	16.3
	Prostate	54.3	37.1
Northeast	Breast	30.8	21.2
	Ovary	30.0	21.6
	Pancreas	30.7	21.4
	Prostate	31.0	22.2
South	Breast	52.0	28.2
	Ovary	35.9	18.4
	Pancreas	49.8	23.5
	Prostate	53.2	27.5
West	Breast	74.8	48.9
	Ovary	41.1	50.2
	Pancreas	56.7	39.2
	Prostate	70.8	55.2
