# Supplementary material for: Disparity in Access to Oncology Precision Care: A Geospatial Analysis of Driving Distances to Genetic Counselors in the U.S
Source: Front Oncol. 2021 Jun 16;11:689927. doi: 10.3389/fonc.2021.689927 (PMC8242948; doi:10.3389/fonc.2021.689927)
Supplement: Supplementary file 9 [file Table_5.pdf]

Table 5. Median and IQR of genetic counselor distance-based access to care (state-level median of distances to the nearest genetic counselor, weighted by cancer incidence rates) for cancer patients by U.S. region (in conventional units and in SI units in square brackets).

Region	Median of access / mi [km]	IQR of access / mi [km]
**Midwest**	35.6 [59.0]	58.5 [94.1]
**Northeast**	16.1 [25.9]	12.1 [19.5]
**South**	34.8 [56.0]	24.6 [39.6]
**West**	64.6 [103.9]	28.2 [45.4]
