# Supplementary material for: Disparity in Access to Oncology Precision Care: A Geospatial Analysis of Driving Distances to Genetic Counselors in the U.S
Source: Front Oncol. 2021 Jun 16;11:689927. doi: 10.3389/fonc.2021.689927 (PMC8242948; doi:10.3389/fonc.2021.689927)
Supplement: Supplementary file 10 [file DataSheet_1.pdf]

Supplementary Material

Supplementary Collection and reconciliation of genetic counselor data

The steps taken to clean and reconcile the data on in-person genetic counselors across the three different sources (NPPES, NSGC, ABGC) are described here. Although data for virtual genetic counselors were available from NSGC, the address data were determined to be unusable for the purposes of deduplication and these data were excluded from the following data deduplication effort.

The NPPES data were downloaded as a flat file as of the May 2020 release and filtered to genetic counselors only as defined by the presence of the Healthcare Provider Taxonomy Code "170300000X" in the first taxonomy code column. Both the ABGC and NSGC data were scraped from the societies' respective public-facing websites and directories using Robotic Process Automation (RPA) as implemented in the software tool UIPath (scraped on 1 July 2020 and 22 June 2020, respectively). For all the data sources data were available on in-person services, and in the NSGC dataset telegenetics services data were available.

Next, (duplicated, unmatched) data were combined on provider name, credentials, specialty, provenance, longitude, latitude, address, city and state from the raw geocoded data into a common data model, retaining any duplications. Records with the same name were flagged for manual human review, and for sets of duplicated names the data were collapsed to one record containing the most complete address information, if the addresses were consistent with each other. In the case where some providers had duplicated names but addresses that were significantly different, each instance of an address was taken as a unique provider. Following an initial review, a second round of human review as conducted to mitigate possibilities of error, and this review included updating addresses and removing incorrectly geocoded addresses. In the end, the merged data represent the most complete data available for each individual genetic counselor and the addresses associated with each genetic counselor representative of the most complete and up-to-date address from all available data sources. The dataset captured all the known genetic counselors from the three data sources used and represents genetic counselors in all 50 states and the District of Columbia who provide in-person genetic counseling services. Genetic counselors working in US territories or military bases were excluded.

The results of this exercise are shown as a Venn diagram in Supplemental Figure 1, which breaks down number of unique genetic counselors by provenance.

Detailed hypothesis test results of disparities

Table 1, Table 2, and Table 3 present respectively the results of: medians and interquartile ranges of access metrics by U.S. region, pairwise Wilcoxon tests for differences in access by U.S. region, medians and interquartile ranges of access by cancer type, pairwise Wilcoxon tests for differences in access by cancer type, medians and interquartile ranges of SDoH metrics by county-level access, as well as Wilcoxon rank sum tests for differences in SDoH by county-level access. An additional analysis of county-level SDoH differences between metropolitan and nonmetropolitan areas showed the same patterns as for the covered versus not covered counties, consistent with the hypothesis that covered counties tend to be more urban.

Differences in access by a combination of both region and cancer type were also analyzed, finding that there are indeed significant differences by this grouping (Kruskal-Wallis p-value of less than 0.001). Supplemental Figure 2 presents the distributions and Table 4 the medians and interquartile ranges of access metrics.

Distance-based definition of access

Distance-based access to care by cancer patients was calculated by taking the weighted median (as implemented in the stats package of R) county-level distance in miles and kilometers to the closest genetic counselor, with weights provided by cancer incidence rate, for all cancer types or by each of the four *BRCA*-associated tumor sites. Physical access to care for cancer patients was systematically different at the 99.9% confidence level between U.S. regions (Kruskal-Wallis *p*-value of less than 0.001), with the most significant disparity existing between the West and Northeast regions (pairwise Wilcoxon Bonferroni-adjusted *p*-value of less than 0.001) and was also different for the selected cancer types. There were also statistically significant differences at the 99% confidence level in access between different types of incident cancer patients (Kruskal-Wallis *p*-value of 0.002), with the most significant difference existing between prostate and ovarian cancer types (pairwise Wilcoxon Bonferroni-adjusted *p*-value of 0.006). Summary statistics and hypothesis test results are presented in Supplemental Table 3.

Analysis of sensitivity to genetic counselor location data source

To address errors that may have arisen in creating an integrated list of U.S.-based genetic counselors across multiple data sources, the full data were subset by source(s) to understand what potential differences may arise in analysis when looking at specific sources of data. Because it was known that data from NPPES were less reliable than data from ABGC and NSGC due to more infrequent culling of inactive records, the data were subset into 1) data from ABGC as the only source (inclusive of duplicate entries from NSGC and NPPES – this set contained 3077 genetic counselors, or 0.95 genetic counselors per 100,000 US residents); 2) data from NSGC as the only source (inclusive of duplicate entries from ABGC and NPPES – this set contained 1975 genetic counselors, or 0.61 genetic counselors per 100,000 US residents); and 3) data from either ABGC or NSGC as a source (inclusive of duplicate entries from NPPES that appeared in one or both data sources – this set contained 3598 genetic counselors, or 1.11 genetic counselors per 100,000 US residents).

For each of the three subsets of data, physical access to care was analyzed using the same methodology as for the full data with calculated drive-times being limited to the closest genetic counselors in each subset of data. SDoH were also analyzed using the same methodology for each subset to understand differences in counties with and without in-person genetic counselor access.

There were statistically significant differences in access to care for cancer patients across U.S. regions, for different types of cancer, and for different types of cancer in different U.S. regions. The pairwise analysis of U.S. regions saw the largest differences between the West and Northeast for all three subsets as well as the full data. Pairwise analysis of cancer types saw the largest differences between prostate and ovarian cancer patients for all three subsets as well as the full data. There were slight differences of magnitude in exact drive-time in each subset, but the overall conclusion that differences in access to care was statistically significant, particularly in the Northeast, which tended to have the best access to care, and for ovarian cancer patients, who tended to have the best access to care.

When looking at SDoH in counties with and without in-person genetic counselor access, there were statistically significant differences in every measure for every subset of the data as well as the full data. Additionally, differences in magnitude were negligible for most measures between the subset data and the full. Overall, analysis of any subset of data led to the same conclusion as with the full data: residents of counties with in-person genetic counselor access are younger, more diverse, have higher incomes, more likely to have health insurance coverage (particularly private insurance), and be better educated. That is, regardless of whether the full data or a subset of the data were examined, counties with in-person genetic counselor access had characteristics associated with metropolitan areas.

Analysis of virtual access at the state level

The patterns of virtual access by genetic counselors were also analyzed at the state level, as reported in the public facing NSGC member directory. As all states had at least one practicing virtual genetic counselor, states were first categorized into high- and low-density states, where density was defined as the number of virtual genetic counselors in a state divided by the population estimate of that state from 2018 ACS 5-year estimate data, without accounting for the possibility that a genetic counselor might practice in additional states that don't require licensure. The distribution of this metric by state was calculated, shown in Supplemental Figure 3, and the distribution mean found to be 2 genetic counselors per 100,000 people. States with fewer than this were labeled as low-density and those with higher labeled as high-density. A similar SDoH analysis as for the county-level in-person genetic counselors was then performed, but at the state level (Supplemental Figure 2). Table 5 shows medians and interquartile ranges of the metrics for the two access groups, as well as results of Bonferroni-corrected Wilcoxon Rank Sum Tests, and Supplemental Figure 4 shows probability density functions of the distributions. The results show that only fraction of a state's population that identifies as white and fraction of a state's population without a high school degree are statistically significantly different between the two groups, and specifically that states with relatively higher densities of virtual genetic counseling access are less diverse and more highly educated. It is interesting to note that there are fewer types of SDoH that are significantly different between the high- and low-access states than there are between the covered and uncovered counties, and that the correlation between proportion of white residents in a state and virtual access is opposite that between proportion of white residents in a county and in-person access. The reason for this inconsistency may be that virtual access is examined at the state level, which could mask intrastate variation present for the in-person access at the county level.

Supplemental Figures

Supplemental Figure 1. Provenance of genetic counselor data: Venn diagram of numbers of unique genetic counselors extracted from each source of provider information.

Supplemental Figure 2. Access to care by patients with *BRCA*-associated cancers by cancer type and U.S. region: box plots of access metric by combination of U.S. Census region and cancer type. Note that the state-level access to care (defined as the median drive time for a cancer patient to a genetic counselor) is plotted on a log axis.

Supplemental Figure 3. Virtual genetic counselor access: histogram of state-level per-capita numbers of virtual counselors and measures of central tendency.

Supplemental Figure 4. State-level SDoH: probability densities of state-level SDoH distributions by mean-split per-capita virtual genetic counselor density.

Supplemental Tables

Table 1. Median and IQR of genetic counselor access to care (state-level median of drive-times to the nearest genetic counselor, weighted by cancer incidence rates) for cancer patients by U.S. region

Table 2. Median and IQR of access to care (state-level median of drive-times to the nearest genetic counselor, weighted by cancer incidence rates) for patients with BRCA-associated cancers by cancer type

Table 3. County-level SDoH by genetic counselor access groups (in-person vs. without in-person). Note that dispersion is represented by the inter-quartile range, which is not sensitive to sample size and hence not a reflection of population variance. (*: >95% confidence; **: >99% confidence; ***:>99.9% confidence, N.S.: < 90% confidence)

Table 4. Median and IQR of access to care for BRCA-associated cancer types in different U.S. regions

Table 5. Median and IQR of genetic counselor distance-based access to care (state-level median of distances to the nearest genetic counselor, weighted by cancer incidence rates) for cancer patients by U.S. region (in conventional units and in SI units in square brackets).
